# SB 206553, a putative 5-HT_2C_ inverse agonist, attenuates methamphetamine-seeking in rats

**DOI:** 10.1186/1471-2202-13-65

**Published:** 2012-06-14

**Authors:** Steven M Graves, T Celeste Napier

**Affiliations:** 1 Department of Pharmacology Rush, University Medical Center, 1735 W Harrison Street, Cohn Research Building, Chicago, IL, 60612, USA; 2 Center for Compulsive Behavior and Addiction, Rush University Medical Center, 1735 W Harrison Street, Cohn Research Building, Chicago, IL, 60612, USA

**Keywords:** Methamphetamine, Serotonin, Self-administration, Motor, Inverse agonist, Seeking

## Abstract

**Background:**

Methamphetamine (meth) dependence presents a substantial socioeconomic burden. Despite the need, there is no FDA-approved pharmacotherapy for psychostimulant dependence. We consider 5-HT_2C_ receptors as viable therapeutic targets. We recently revealed that the atypical antidepressant, mirtazapine, attenuates meth-seeking in a rodent model of human substance abuse. Mirtazapine historically has been considered to be an antagonist at 5-HT_2C_ receptors, but more recently shown to exhibit inverse agonism at constitutively active 5-HT_2C_ receptors. To help distinguish the roles for antagonism *vs.* inverse agonism, here we explored the ability of a more selective 5-HT_2C_ inverse agonist, SB 206553 to attenuate meth-seeking behavior, and compared its effects to those obtained with 5-HT_2C_ antagonists, SDZ Ser 082 and SB 242084. To do so, rats were trained to self-administer meth and tested for seeking-like behavior in cue reactivity sessions consisting of contingently presenting meth-associated cues without meth reinforcement. We also explored motor function to determine the influence of SB 206553 and SDZ Ser 082 on motor activity in the presence and absence of meth.

**Results:**

Like mirtazapine, pretreatment with SB 206553 (1.0, 5.0, and 10.0 mg/kg), attenuated meth-seeking. In contrast, the antagonists, SDZ Ser 082 (0.1, 0.3, and 1.0 mg/kg) and SB 242084 (3.0 mg/kg) had no effect on cue reactivity (CR). SB 242084 (3.0 mg/kg) failed to attenuate the effects of 5.0 and 10 mg/kg SB 206553 on CR. Motor function was largely unaltered by the 5-HT_2C_ ligands; however, SB 206553, at the highest dose tested (10.0 mg/kg), attenuated meth-induced rearing behavior.

**Conclusions:**

The lack of effect by 5-HT_2C_ antagonists suggests that meth-seeking and meth-evoked motor activity are independent of endogenous 5-HT acting at 5-HT_2C_ receptors. While SB 206553 dramatically impacted meth-evoked behaviors it is unclear whether the observed effects were 5-HT_2C_ receptor mediated. Thus, SB 206553 deserves further attention in the study of psychostimulant abuse disorders.

## Background

Psychostimulants such as cocaine and methamphetamine (meth) dramatically enhance transmission of monoamines, including serotonin (5-HT). It is clear that the 5-HT_2C_ receptor subtype is involved in stimulant-mediated behaviors [1,2]. For example, 5-HT_2C_ receptor agonists decrease cue-induced reinstatement of cocaine-seeking [3-6] (5-HT_2C_ antagonists have no effect [5,7]). These latter findings suggest that cue-associated seeking behavior is not dependent on 5-HT levels at synapses expressing 5-HT_2C_ receptors (illustrated by the lack of effect of antagonists on cocaine cue-induced reinstatement); nonetheless, the systems regulating seeking behavior are under negative control by activated 5-HT_2C_ receptors (illustrated by the ability of 5-HT_2C_ agonists to decrease cue-induced reinstatement of cocaine-seeking). The majority of behavioral studies focusing on 5-HT_2C_ receptors have relied on agonist/antagonist relationships. However, evidence indicates constitutive activity (i.e. agonist-independent activation) of 5-HT_2C_ receptors *in vivo*[8-10] and it currently is unknown how constitutively active 5-HT_2C_ receptors impact stimulant-induced behaviors.

5-HT_2C_ receptors are the only known G protein coupled receptor to undergo mRNA editing by adenosine deaminases [11,12]; a process that has important implications for the level of constitutive activity. Adenosine to inosine switches result in amino acid substitutions in the second intracellular loop of the receptor protein [13-15]. The unedited 5-HT_2C_ receptor displays high levels of constitutive activity [16-20], and these receptor protein substitutions decrease constitutive activity. Constitutive activity can be attenuated pharmacologically by inverse agonists, these drugs stabilize the receptor in its inactive conformation [21,22]. For example, systemic administration of the 5-HT_2C_ inverse agonist SB 206553 (SB206) enhances dopamine (DA) efflux in the nucleus accumbens and striatum in rats [8], whereas an agonist either decreases or has no effect on accumbal and striatal DA [8,23-27]. Supporting a role of constitutive activity in this effect, the SB206-induced effects were verified to occur independent of endogenous 5-HT [8]. We previously revealed that the atypical antidepressant, mirtazapine, attenuates methamphetamine (meth)-induced sensitization [28], place conditioning [29-31], and seeking [31] in rodent models of human substance abuse. Mirtazapine historically has been considered to be an antagonist at 5-HT_2C_ and other receptors [32-34]; however, more recent studies indicate its action as an inverse agonist at constitutively active 5-HT_2C_ receptors [35,36]. These new observations raised the following questions: Would a more selective 5-HT_2C_ receptor inverse agonist, like mirtazapine, also reduce meth-seeking? A related question is whether seeking for meth is similar to, or differs from, that for cocaine with regard to sensitivity to selective 5-HT_2C_ inverse agonists and/or antagonists?

To answer the above questions, we used two 5-HT_2C_ antagonists of different chemical structures, SB 242084 (SB242) and SDZ Ser 082 (SDZ) as well as the putative 5-HT_2C_ receptor inverse agonist SB206. Dose–response evaluations on meth-seeking behaviors were made for these drugs in using cue reactivity (CR) to meth-associated cues established during a self-administration protocol. We have previously demonstrated that CR offers an effective means to study drug-seeking that, as is more typical in the human scenario, does not depend on prior extinction training [31]. We also revealed that the ability of mirtazapine to alter seeking behavior is similar for CR and cue-induced reinstatement (following extinction) protocols [37]. Finally, to ascertain whether 5-HT_2C_ receptor compounds influenced motor activity, rats tested for CR also underwent motor assessments with acute 5-HT_2C_ ligand pretreatment.

## Methods

### Subjects

Fifty male Sprague–Dawley rats were purchased from Harlan (Indianapolis, IN), acclimated to housing in our local vivarium for 5 days, and handled a minimum of 3 times prior to surgery. Food and water were provided *ad libitum* throughout the study. Rats were maintained in accordance with the Guide for Care and Use of Laboratory Animals (National Research Council, Washington DC) and with the guidelines and approval of the Rush University Institutional Animal Care and Use Committee.

### Drugs

(+)-Methamphetamine HCl (Sigma, St. Louis, MO) was dissolved in sterile saline. The stimulant was self-administered at 0.1 mg/kg/0.1 mL infusion intravenously (iv) and non-contingently administered intraperitoneally (ip) for motor assessments as 1.0 mg/kg. SDZ Ser 082 (SDZ; Tocris, Ellisville, MO), a 5-HT_2C_ antagonist, was dissolved in saline and injected ip (0.1, 0.3, and 1.0 mg/kg). SB 242084 (SB242; Tocris), also a 5-HT_2C_ antagonist, was dissolved in 8 % β-cyclodextran and 1 % citric acid in deionized water and injected ip (3.0 mg/kg). SB 206553 (SB206) (Tocris), a 5-HT_2C_ inverse agonist, was dissolved in 1 % lactic acid in deionized water and injected ip (1.0, 5.0, and 10.0 mg/kg). Serotonin_2C_ ligands were administered in volumes of 1.0 ml/kg with the exception of 10.0 mg/kg SB206, which was administered at 2 ml/kg (from a 5.0 mg/ml stock solution). All drugs were administered as the base. pK_i_ values of SDZ, SB242 and SB206 for 5-HT_2_ receptor subtypes are provided in Table 1.

**Table 1:** **5-HT**_**2C**_**receptors**

	**5-HT**_**2A**_	**5-HT**_**2B**_	**5-HT**_**2C**_
SDZ Ser 082	6.3	6.7	8.1
SB 242084	6.1	6.8	8.2
SB 206553	5.6	7.7	7.8

pK_i_ values (nM) for 5-HT_2_ receptor ligands in HEK-293 or CHO-K1 cells expressing human recombinant 5-HT_2_ receptors [45].

### Surgical procedures

Rats were instrumented with a jugular vein catheter under continuous isoflurane anesthesia. Custom built catheters were constructed with silastic tubing (0.3 mm i.d. x 0.64 mm o.d.; Dow Corning Co., Midland, MI) and implanted into the right jugular vein. The distal end of the catheter extended to the mid-scapular region with a metal guide canulae (22 gauge; Plastics One Inc., Roanoke, VA) and anchored to a plastic mesh. Rats were allowed to recover for a minimum of 5 days prior to beginning self-administration procedures. During this time, rats were handled, inspected and weighed daily to ensure that surgical wounds were healing properly (without infection), that normal behavioral grooming/eating patterns were retained, and that preoperative weight was regained.

### Self-Administration acquisition

**Figure 1:**
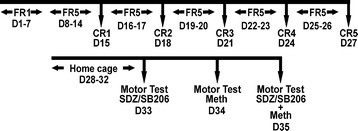
**Timeline of experimental protocol.** Rats self-administered methamphetamine (meth) for 14 consecutive days (3 hrs/day). Days (D) 1–7 a fixed ratio (FR) 1 schedule of reinforcement was used for training. To increase responding rates, an FR5 was employed for days 8–14. On day 15, rats were acclimated to the cue reactivity (CR) protocol. Assessments of 5-HT_2C_ ligands on meth-seeking were made days 18, 21, 24, and 27 (i.e., CR2-CR5); treatments for CR were randomized. Between each CR test, rats were allowed to self-administer meth for two consecutive days on an FR5 schedule (3 hrs/day). Five days after CR5 (on day 33), motor assessments were initiated.

Rats were trained to self-administer meth 3 hr/day for 14 consecutive days in standard operant chambers enclosed in ventilated, sound attenuating cabinets (Med-Associates, St. Albans, VT). Each operant chamber contained two levers; the left lever assigned as the “active” lever and the right the “inactive” lever. Above each lever was a cue light and located on the opposite wall was an in-house light. A cue light above the active lever was activated in association with the infusion pump and the in-house light was subsequently activated for 20s, indicating a time-out period during which responses had no programmed consequences. Responding on the inactive lever also had no programmed consequences. On days 1–7, rats self-administered meth for 3 hr/day on a fixed ratio (FR) 1 schedule of reinforcement. On days 8–14, rats self-administered on a FR5 for 3 hr/day (refer to Figure 1) to enhance lever pressing behaviors and resistance to extinction [38]. Starting on day 8, rats were acclimated to injection procedures *via* daily ip vehicle pretreatments (30 min). The number of active lever presses, inactive lever presses, and infusions were recorded for all sessions. Stable self-administration behavior was operationally defined as <15 % infusion variability between days 13 and 14; failure to reach this criterion resulted in subject exclusion from the study.

### Cue reactivity testing

Meth-seeking behavior was assessed *via* CR tests. CR testing consisted of single, 1 hr extinction sessions during which cues (i.e., cue light, time-out house light, and activation of infusion pump) were contingently presented on an FR1 schedule. During CR tests, infusion lines were filled with sterile saline and remained connected to saline-filled syringes (syringes were disconnected from pumps to prevent excessive fluid intake). Meth was not accessible during CR testing. The number of active and inactive lever presses was measured continuously and tallied in 15 min intervals. On day 15, rats were given a 30 min pretreatment of vehicle and tested for baseline meth-seeking (CR1); baseline was used to equally distribute rats into treatment groups. Rats were tested for an additional four CR tests (CR2-5); between each CR test, rats were allowed to self-administer meth for 3 hr/day on an FR5 schedule (referred to as *intermittent self-administration*; see Figure 1) to prevent extinction training. Rats failing to administer at least 50 % of the number of infusions administered on day 14, for two consecutive days during intermittent self-administration sessions, were removed from the study. Rats were tested for CR after a 30 min pretreatment of 0.1, 0.3, 1.0 mg/kg SDZ and vehicle (treatment group 1) or 1.0, 5.0, 10.0 mg/kg SB206, and vehicle (group 2). The 5-HT_2C_ receptor antagonist, SB242, was tested against the 5-HT_2C_ receptor inverse agonist SB206 in the same rats, wherein SB242 (3.0 mg/kg ip) was administered 45 min, and SB206 (5.0 or 10.0 mg/kg ip) was administered 30 min, prior to the onset of CR testing. Dose order was randomized for all treatment groups. Doses of SB206 and SB242 were guided by literature demonstrating significant neurochemical effects in the nucleus accumbens [8]. Doses of SDZ were selected based on reports revealing an enhancement of the interoceptive cues of cocaine [39] and cocaine-evoked motor activity in naïve rats [40]. We have previously shown that the described CR paradigm is comparable to cue-induced reinstatement and that pharmacological intervention paradigms similar to those tested in the current report similarly reduce CR and cue-induced reinstatement [37]. Assessments were made at an early stage of withdrawal (24 hr) based on our aforementioned work [37] as well as evidence indicating that psychostimulant administration dysregulates neuronal function in the nucleus accumbens shell (a region with constitutively active 5-HT_2C_ receptors [10]) as early as 1–3 days of withdrawal [41] as well as biochemical indices indicating psychostimulant-induced plasticity in multiple brain regions during early phase withdrawal (1–3 days) using place conditioning paradigms [42,43] and motor sensitization [44].

### Motor assessments

A subset of rats tested for CR dose–response assessments was used to determine the motor effects of SDZ (1.0 mg/kg ip) and SB206 (5.0 and 10.0 mg/kg ip) in the presence and absence of meth (1.0 mg/kg ip). For this study, rats were withdrawn from meth and remained in their home cages during protocol days 28–32 and received no treatment (refer to Figure 1). After this 5 day period, rats were tested for motor activity for 3 consecutive days (days 33–35). All motor assessments were conducted using automated small animal activity boxes (Accuscan Instruments, Columbus, OH) equipped with two banks of photobeams positioned at different heights to characterize motor activity in three dimensional space. Rats were habituated to activity chambers for 1 hr prior to each motor test. On day 33, rats were administered 1 ml/kg of the respective vehicle for each test drug (rats for effects of SDZ were administered saline and rats tested for SB206-induced effects were administered 1 % lactic acid in deionized water). The injected rats were immediately returned to motor boxes for 1 hr after which rats were injected with either SDZ (1.0 mg/kg), or SB206 (5.0 or 10.0 mg/kg) and behavior was recorded for an additional 1 hr. Motor data collected 30 min post-injection were subsequently analyzed; this time frame reflected the one that was relevant to CR behaviors. On day 34, rats were administered a 30 min pretreatment of vehicle (saline or 1 % lactic acid), then administered 1 mg/kg meth (ip) and behavior recorded for 1 hr. On day 35, the procedure from day 34 was repeated using SDZ (1.0 mg/kg), or SB206 (5.0 or 10.0 mg/kg) (ip) instead of respective vehicles. Peak meth effects occurred 15 min post meth injection; meth-evoked motor activity was therefore analyzed for the last 45 min of testing (i.e., 15 min post meth injection). Horizontal activity (number of beam breaks in the horizontal plane), vertical activity (number of beam breaks in the vertical plane indicating rearing-like behavior), and total distance (cm traversed within the chamber) were recorded. These assessments provide a reliable index of overall motor patterns evoked by this dose of meth [28]. Stereotypy (rapid, repetitive behaviors) is a prominent component of meth-induced motor activity [28]; therefore, stereotypy number (the number of beam breaks repetitively disrupted) also was analyzed for meth-evoked motor activity. Rats tested for effects of SDZ on motor function were also tested for SDZ effects on CR; similarly, rats tested for effects of SB206 on motor function had prior exposure to SB206 during CR assessments.

### Statistical analysis

Active lever presses, inactive lever presses, and number of infusions achieved during self-administration sessions were analyzed using a one-way rmANOVA with Newman-Keuls *post-hoc* analysis. The differences in lever pressing during CR tests were detected using two-way rmANOVA with Newman-Keuls *post-hoc* analysis. Lever pressing behavior for days 16 and 26 of intermittent self-administration, and motor activity were analyzed using a paired *t*-test. For all tests, α = 0.05. Data are presented as mean ± SEM. Data greater than two standard deviations from the mean were considered to be statistical outliers and were excluded from the analyses; for CR, outliers were determined from behavior collapsed across the first 30 min of the tests.

## Results

### Stable meth self-administration

**Figure 2:**
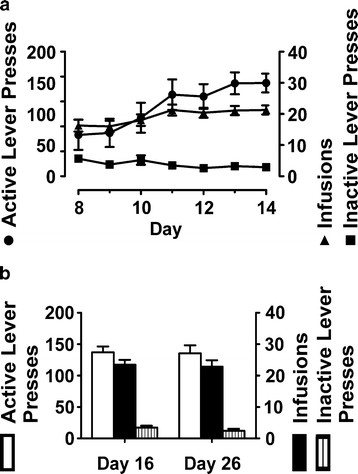
**Acquisition and maintenance of methamphetamine self-administration.** Illustrated is methamphetamine (meth) self-administered as 0.1 mg/kg/0.1 ml on a fixed ratio (FR) 5 schedule of reinforcement. Forty rats completed the paradigm and were tested for cue reactivity (CR). (**A**) Protocol days 8–14; rats demonstrated stable self-administration. As measured by one-way rmANOVA, no significant differences occurred across the last 3 days of self-administration (days 12–14) for active lever presses (F_(3,37)_ = 8.95; two statistical outliers removed), inactive lever presses (F_(3,35)_ = 4.94; four statistical outliers removed), or infusions (F_(3,37)_ = 12.46; two statistical outliers removed). (**B**) Protocol days 16 and 26. Between each cue reactivity (CR) assessment, rats were allowed to self-administer for two consecutive days on an FR5 schedule for 3 hr/day. Using paired *t*-tests, no differences occurred between days 16 and 26 for active lever pressing (p = 0.89; one statistical outlier removed), inactive lever pressing (p = 0.23; three statistical outliers removed) or number of infusions received (p = 0.76; one statistical outlier removed).

Fifty rats acquired the self-administration task; four rats were removed from the study as they displayed >15 % infusion variability between the last two training sessions (i.e., days 13 and 14); three rats died after a self-administration session. An additional 3 rats were removed due to failure to maintain stable self-administration behavior during periods of intermittent self-administration (i.e., responding for two consecutive days fell below 50 % of the number of reinforcements received on day 14 of self-administration). Of the 40 rats that completed the study, there were no differences for active lever pressing (p = 0.89; one statistical outlier removed), inactive lever pressing (p = 0.23; three statistical outliers removed), or infusions (p = 0.76; one statistical outlier removed) for days 11–14 (paired *t*-test; Figure 2A).

### Meth-seeking behavior: cue reactivity

A within-subjects design was used wherein each rat was tested with three doses (randomized) of either the antagonist (SDZ; n = 11), inverse agonist (SB206; n = 8) and the respective vehicles. Similarly, a within-subjects design was used to test for antagonist/inverse agonist interactions with 3.0 mg/kg SB242 *vs*. 10.0 (n = 11) and 5.0 mg/kg (n = 10) SB206, and respective vehicles. There were no significant differences among rats assigned to SDZ (treatment group 1), SB206 (treatment group 2), or interaction studies (treatment groups 3 and 4) for active or inactive lever pressing during CR1 (ANOVA, p > 0.05; data not shown). Additionally, active lever pressing comparing CR after vehicle pretreatment was not different among the four treatment groups (ANOVA, p > 0.05; data not shown). Self-administration behavior was not altered by the interposed CR testing sessions; the first and last day of intermittent self-administration were not significantly different for active lever presses, inactive lever presses, or infusions comparing day 16 and day 26 (paired *t*-test, p > 0.05; Figure 2B). In agreement with our prior report validating consistent self-administration and seeking [37], these data verify that behavior is persistent throughout the paradigm.

**Figure 3:**
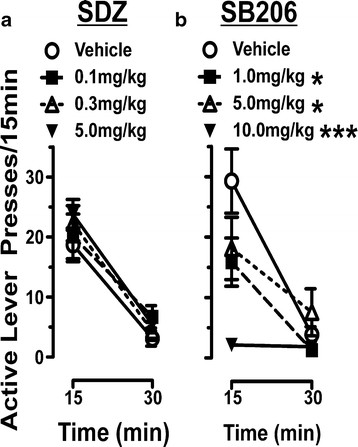
**SB206 reduced reactivity to methamphetamine-associated cues.** Shown are active lever presses in 15 min intervals for the first 30 min of cue reactivity (CR) testing as exhibited by rats that completed dose–response assessments of SDZ Ser 082 (SDZ; n = 10) and SB 206553 (SB206; n = 8). The x-axis legend refers to time elapsed after treatment and immediately placing the subject into the operant chamber; data represent total active lever presses between 30-45 min and 45-60 min after treatment. One statistical outlier was removed from the SDZ treatment group. (**A**) SDZ had no effect on active lever pressing at any dose tested (two-way rmANOVA). (**B**) SB206 significantly decreased active lever presses in the first 15 min of testing at 1.0, 5.0, and 10.0 mg/kg (*p < 0.05 comparing vehicle *vs*. 1.0 and 5.0 mg/kg SB206; ***p < 0.001 comparing vehicle *vs*. 10.0 mg/kg SB206; Newman-Keuls *post-hoc*).

**Figure 4:**
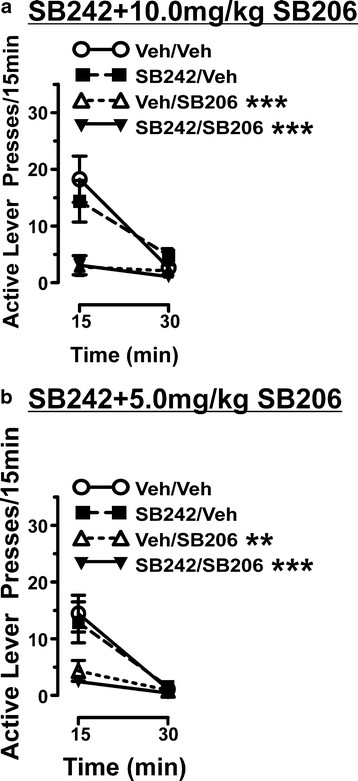
**Attenuation of active lever pressing by SB206 was not reversed by the 5-HT**_**2C**_**antagonist SB242.** Shown are active lever presses in 15 min intervals for the first 30 min of cue reactivity (CR) testing from rats that completed interaction studies with 3.0 mg/kg SB 242084 (SB242) *vs*. 10.0 mg/kg SB 206553 (SB206; n = 11) or 5.0 mg/kg SB206 (n = 9; one statistical outlier removed). Rats were tested after a pretreatment of vehicle or 3.0 mg/kg SB242, and vehicle, 5.0, or 10.0 mg/kg SB206. The x-axis legend refers to time elapsed after treatment and immediately placing the subject into the operant chamber; data represent total active lever presses between 30-45 min and 45-60 min after treatment. (**A**) 5-HT_2C_ antagonism with 3.0 mg/kg SB242 had no effect on CR when administered alone and did not block the effects of 10.0 mg/kg SB206. SB206 significantly decreased active lever presses in the first 15 min of testing (***p < 0.001; Newman-Keuls comparing veh/veh *vs*. 10.0 mg/kg SB206) and significance was retained even following pretreatment with SB242 (***p < 0.001; comparing veh/veh *vs*. SB242/SB206). (**B**) 5-HT_2C_ antagonism with 3.0 mg/kg SB242 had no effect on active lever pressing when administered alone and did not block the effects of 5.0 mg/kg SB206. SB206 significantly decreased active lever presses in the first 15 min of testing (**p < 0.01; Newman-Keuls comparing veh/veh *vs*. 5.0 mg/kg SB206) and significance was retained following pretreatment with SB242 (***p < 0.001 comparing veh/veh *vs*. SB242/SB206).

Independent of the CR test number or pretreatment, meth-seeking behavior occurred most intensely within the first 15 min of CR testing (Figures 3 and 4) as previously reported [37]; by 30 min, levels emulated those expressed on the inactive lever (data not shown). It is also important to note that the number of lever presses achieved during self-administration sessions are far greater than the number during CR tests with a vehicle pretreatment. This is a consequence of i) lever presses during CR tests are not reinforced with meth and ii) self-administration sessions are 3 hrs long whereas CR tests are shown in15min intervals.

SDZ, the 5-HT_2C_ antagonist, had no effect on number of active (Figure 3A) or inactive lever pressing (data not shown) at any dose tested. Two-way rmANOVA comparing active lever pressing revealed no significant Treatment effect (F_(3,36)_ = 0.81), a significant Time effect (F_(1,36)_ = 97.57), and no Treatment x Time interaction (F_(3,36)_ = 0.49). In contrast, administration of the 5-HT_2C_ inverse agonist SB206 attenuated active lever pressing in the first 15 min at all doses tested (Figure 3B) resulting in an approximately 25 %, 50 %, and 95 % reduction in active lever pressing by 1.0, 5.0, and 10.0 mg/kg, respectively. Two-way rmANOVA revealed a significant effect of Treatment (F_(3,24)_ = 5.97) and Time (F_(1,24)_ = 34.70), and a Treatment x Time interaction (F_(3,24)_ = 5.80). Inactive lever pressing was attenuated by approximately 60, 80, and 100 % for 1.0, 5.0, and 10.0 mg/kg SB206, respectively, in the first 15 min of testing (data not shown). While the percentage change in inactive lever pressing appears dramatic, this only corresponds to approximately 2, 3, and 4 fewer inactive lever presses by 1.0, 5.0, and 10.0 mg/kg, respectively.

Similar to the effects seen with SDZ, 3.0 mg/kg of SB242 (also a 5-HT_2C_ receptor antagonist) had no effect on active (Figure 4) or inactive (data not shown) lever pressing when administered alone. Pretreatment with 3.0 mg/kg SB242 15 min prior to administration of 10.0 or 5.0 mg/kg of SB206 however, did not attenuate the SB206-induced suppression of active (Figure 4) and inactive (data not shown) lever pressing. Two-way rmANOVA revealed a significant Treatment effect (F_(3,40)_ = 6.04), Time effect (F_(1,40)_ = 21.97), and Treatment x Time interaction (F_(3,40)_ = 7.36) comparing the effects of 3.0 mg/kg SB242 and 10.0 mg/kg SB206 as well as a significant Treatment effect (F_(3,32)_ = 4.90), Time effect (F_(1,32)_ = 36.71), and Treatment x Time interaction (F_(3,32)_ = 5.151) comparing the effects of 3.0 mg/kg SB242 and 5.0 mg/kg SB206. Our antagonist data are consistent with the inability of 5-HT_2C_ antagonism to alter cue-induced reinstatement of cocaine-seeking [5,7]. However, because the SB242 did not prevent SB206-induced decreases in lever pressing, it is unclear whether SB206 effects are 5-HT_2C_ dependent.

### Normal exploratory and meth-evoked motor activity

To assess the ability for the 5-HT_2C_ ligands to impact non-operant-related motor activity, SDZ and SB206 were tested for motor effects in the absence and presence of meth. In the absence of meth, neither the SDZ (1.0 mg/kg) nor SB206 (5.0 or 10.0 mg/kg) altered motor activity (Table 2). SDZ (1.0 mg/kg) had no effect on meth-evoked motor activity, (Table 3). Likewise, the lower dose of 5.0 mg/kg SB206, which was sufficient to attenuate meth-seeking, had no effect on meth-evoked motor activity (Table 3). The higher dose (10.0 mg/kg) only significantly attenuated meth-evoked vertical activity.

**Table 2:** Effects of SDZ Ser 082 and SB 206553 on motor activity

	**Horizontal Activity**	**Total Distance**	**Vertical Activity**
Vehicle	727 ± 85	189 ± 66	46 ± 10
1.0 mg/kg SDZ	572 ± 164	182 ± 65	88 ± 32
Vehicle	634 ± 66	186 ± 63	37 ± 14
5.0 mg/kg SB206	564 ± 132	107 ± 34	9 ± 3
Vehicle	379 ± 97	82 ± 42	32 ± 11
10.0 mg/kg SB206	453 ± 41	132 ± 30	11 ± 5

Rats that had undergone repeated cue reactivity testing were withdrawn from methamphetamine and assessed for motor effects after pretreatment with 1.0 mg/kg SDZ Ser 082 (SDZ; 5-HT_2C_ receptor antagonist), 5.0 mg/kg SB 206553 (SB206; putative 5-HT_2C_ receptor inverse agonist), 10.0 mg/kg SB206 and respective vehicles. Serotonin_2C_ receptor antagonism and inverse agonism had no effect on motor activity.

**Table 3:** Meth-evoked motor activity: SDZ Ser 082 and SB 206553 effects

	**Horizontal Activity**	**Total Distance**	**Vertical Activity**	**Stereotypy Count**
Vehicle	8797 ± 738	3832 ± 336	1142 ± 122	477 ± 33
1.0 mg/kg SDZ	8598 ± 858	3723 ± 392	996 ± 190	460 ± 29
Vehicle	7448 ± 1025	3113 ± 444	797 ± 141	446 ± 29
5.0 mg/kg SB206	9067 ± 711	4326 ± 572	594 ± 120	471 ± 18
Vehicle	8429 ± 1133	3705 ± 692	1124 ± 201	471 ± 26
10.0 mg/kg SB206	6448 ± 1276	2245 ± 499	398 ± 91**	394 ± 58

Rats that had undergone repeated cue reactivity testing were withdrawn from methamphetamine and assessed for methamphetamine-evoked (1.0 mg/kg) motor effects after a 30 min pretreatment with 1.0 mg/kg SDZ Ser 082 (SDZ; 5-HT_2C_ receptor antagonist), 5.0 mg/kg SB 206553 (SB206; putative 5-HT_2C_ receptor inverse agonist), 10.0 mg/kg SB206 and respective vehicles. Serotonin_2C_ receptor antagonism had no effect on methamphetamine-evoked motor activity whereas inverse agonism with 10.0 mg/kg SB206 significantly attenuated vertical activity (paired *t*-test; **p < 0.01).

## Discussion

### SB 206553 attenuates methamphetamine-seeking

Results from the current study are the first to investigate the role of 5-HT_2C_ receptor activity in meth-mediated behaviors and the first to test SB206 in a rodent model of addiction. We revealed that the putative inverse agonist robustly and dose-dependently attenuated meth-seeking without impacting exploratory motor activity at 5.0 mg/kg and 10.0 mg/kg. The 5-HT_2C_ antagonists had no effect on either meth-seeking or meth-induced motor activity. These results suggest that our prior work with mirtazapine may reflect the ability of mirtazapine to act as an inverse agonist, not as a 5-HT_2C_ antagonist.

The finding that the antagonist SB242 did not attenuate the effects of the inverse agonist SB206 was unexpected, and raised the question regarding the receptor selectivity of SB206. SB206 has high affinity for both the 5-HT_2C_ and 5-HT_2B_ receptors (pK_i_ = 8.5 and pK_i_ = 8.26, respectively in HEK-293 or CHO-K1 cells expressing human recombinant 5-HT_2C_ or 5-HT_2B_ receptors; Table 1) [45] with 100 fold or greater selectivity over other receptor targets including 5-HT_2A_ receptors [46]. In the current study, SB206 may antagonize 5-HT_2B_ receptors, which are known to regulate behavioral and DA-enhancing effects of amphetamine [47]. However, the increase in accumbal DA seen with 5.0 mg/kg SB206 is not altered with coadministration of the 5-HT_2B_ antagonist LY 266097 indicating that 5.0 mg/kg SB206 does not substantially antagonize 5-HT_2B_ receptors [47].

Antagonism of 5-HT_2A_ receptors are a third 5-HT receptor that may be contributing to our findings. 5-HT_2A_ antagonism with M100907 attenuates cue-induced reinstatement of cocaine-seeking [48] and meth-seeking (unpublished data). As mentioned above, SB206 demonstrates 100 fold selectivity over the 5-HT_2A_ receptor. 5-HT_2A_ antagonism by SB206, particularly at 10.0 mg/kg, is plausible but unlikely at 1.0 and 5.0 mg/kg, for amphetamine-evoked DA concentrations in the nucleus accumbens and striatum are attenuated by 5-HT_2A_ antagonism but 5.0 mg/kg SB206 has no effect [49] suggesting that at this dose, SB206 does not act on 5-HT_2A_ receptors. Additionally, 5.0 mg/kg SB206 and 5-HT_2A_ antagonism with SR 46349B have oppositional effects demonstrated by striatal ^11^ C]raclopride binding lending further evidence to the selectivity of SB206 for 5-HT_2C_ over 5-HT_2A_ receptors [50]. Taken together, the available *in vivo* data suggests that SB206 does not antagonize 5-HT_2A_ receptors at 5.0 mg/kg.

SB206 may also act as a positive allosteric modulator of the α7 nicotinic acetylcholine receptor with an EC_50_ of 1.5 μM for potentiation of responses evoked in GH4C1 cells by EC_20_ nicotine [51]. Dunlop et al., find that 3, 10, and 30 mg/kg SB206 reverses MK-801-induced deficits in prepulse inhibition, a classical model of schizophrenia, but only confirm actions as a positive allosteric modulator using a nicotinic antagonist against 10.0 mg/kg SB206. It is unclear whether lower doses, such as 1.0 and 5.0 mg/kg SB206, would also act as a positive allosteric modulator at α7 nicotinic receptors. Consistent with our findings, antagonism of α7 nicotinic receptors in the ventral tegmental area attenuates the reward-facilitating effect of cocaine [52] whereas nicotine administration attenuates reinstatement of meth-seeking [53]. The role of α7 nicotinic receptors and in particular, positive allosteric modulation of this receptor, in meth abuse is understudied. Future investigation are needed to determine if this receptor is engaged by SB206 in the dose range tested before actions at the α7 receptor can be ruled out.

Debating receptor specificity based on our findings is difficult; however, it is important to note that a prior report showed that 1.0 mg/kg SB242 attenuates 5.0 mg/kg SB206-induced increases in striatal and accumbal DA [8]. Reasons why Spampinato and colleagues were able to antagonize SB206 effects and we were not may relate to the treatment history of rodent subjects. In the aforementioned neurochemical study [8], subjects were stimulant naïve, halothane anesthetized rats. In our study, rats had an extensive history of meth self-administration. This chronic meth history may have modified the 5-HT_2C_ system, for we have observed a functionally upregulated response to systemic 5-HT_2A/C_ receptor agonism in the ventral pallidum of rats chronically administered meth [54]. Repeated meth administration may result in changes in mRNA editing profiles (and thus, levels of constitutive activity). Proof of concept for this hypothesis is seen with studies on stress, antidepressant, and antipsychotic administration where mRNA editing of the 5-HT_2C_ receptor is altered [55-58]. In addition to changing constitutive activity, mRNA editing of the 5-HT_2C_ receptor also alters the binding affinity of ligands whereby increased editing most often results in decreased agonist affinity for the receptor with enhanced or no effect on antagonists and inverse agonist binding affinity [16-18,20]. It is unknown how editing patterns affect the affinities of SB206 and SB242. Thus, the inability of SB242 to augment the effects of SB206 on CR may reflect changes in affinity for constitutively active isoforms and/or in the allosteric constant (i.e., an index of the concentration of constitutively active *vs*. non-constitutively active receptors). Unfortunately *ex vivo* binding studies, which would greatly facilitate our studies, are hampered by a lack of available tritiated ligands with 5-HT_2C_ specificity. While future studies are needed to explore the mechanism of action for SB206 and determine receptor specificity *in vivo*, we speculate that despite a lack of antagonism by SB242, 5-HT_2C_ receptors may still be involved. Moreover, continued study is necessary to determine the effects of 5-HT_2C_ receptor ligands on neuron function; to provide further insights our laboratory is conducting patch clamp studies in *ex vivo* slice preparations to determine the effects of 5-HT_2C_ ligands, including SB206, on neurophysiology.

### SB 206553, but not SDZ Ser 082, attenuates methamphetamine-evoked motor activity

Acute antagonism of 5-HT_2C_ receptors enhances the motor effects of acute cocaine administration; however, this enhancement is lost in rats that are motorically sensitized by repeated injections of cocaine [40]. This is comparable to our current findings wherein rats with a history of meth self-administration, 5-HT_2C_ antagonism did not alter meth-evoked motor activity in rats. A single study testing SB206 on cocaine-evoked motor activity finds an attenuation of cocaine-evoked motor activity by 1.0 mg/kg SB206 but a potentiation with 4.0 mg/kg [59]; however, this was an acute study with a single injection of cocaine making it difficult to compare with the current investigations using meth self-administration. In rats with a history of meth self-administration, only meth-evoked vertical activity was attenuated by 10.0 mg/kg SB206. Both our findings and [59] are in agreement in that SB206 does not alter normal exploratory motor behavior suggesting that SB206 is not sedating; differences in our study and [59] regarding the ability to augment psychostimulant-evoked motor activity may reflect behavioral differences consequent to meth-induced plasticity. This hypothesis is supported by [40] and our current findings wherein 5-HT_2C_ antagonism fails to augment cocaine-evoked and meth-evoked motor activity in rats with a history of repeated cocaine [40] or meth administration.

### SB 206553 decreases inactive lever pressing

Antagonists had no effect, whereas SB206 attenuated inactive lever pressing. Mirtazapine, also an inverse agonist at the 5-HT_2C_ receptor [35,36], similarly attenuates inactive lever pressing in a cue-induced reinstatement paradigm without effecting non-operant related motor activity in a small animal activity chamber in rats with a history of meth self-administration or rotorod performance in naïve rats [37]. Moreover, in naïve rats, administration of 1, 3, and 4 mg/kg SB206 has no effect on motor activity (measured in activity chambers) [59]. A similar pattern is found for GABA_B_ receptor activation. The GABA_B_ agonist (CGP44542) decreases inactive lever pressing in an operant task [60], yet has no effect on performance in an intracranial self-stimulation procedure [61]. In summary, as SB206 had no effect on motor activity in in rats with a meth history (current report) or in naïve rats [59], and that mirtazapine, also an inverse agonist at 5-HT_2C_ receptors [35,36], does not impair motor activity in naïve rats [37], we considered that decreased lever pressing of both active and inactive levers seen in the current study was not a consequence of decreased motor activity. Accordingly, we propose that SB206 blunted the *salience* of the levers, both active and inactive, rather than impairing the ability of the rats to perform the operant task. This interpretation is supported by our prior demonstration that significance of meth-associated context cues are reduced by mirtazapine [31]. Additional studies aimed at exploring the effect of SB206 on non-drug reinforced behaviors may advance this hypothesis; however, as 5-HT_2C_ receptors regulate food intake [6,62,63] as well as intracranial self-stimulation behavior [64,65], such evaluations may challenging to conduct and difficult to interpret.

## Conclusions

The presented findings are the first to explore the effects of 5-HT_2C_ antagonists and putative inverse agonist, SB206, on meth-seeking and meth-induced motor activity. Serotonin_2C_ antagonism had no effect on meth-seeking or motor activity (in the presence or absence of meth); in contrast, SB206, attenuated meth-seeking. This effect was selective, as SB206 agonist did not alter exploratory behavior, and only the higher dose reduced one motor parameter evoked by meth (i.e., vertical activity). There is a growing interest in assessing the role of constitutively active 5-HT_2C_ receptors *in vivo*. It is currently unclear whether SB206 elicits the observed effects *via* 5-HT_2C_ receptor inverse agonism; nonetheless, based on the dramatic reduction in seeking behavior, further investigation of this compound is merited as well as continued study of 5-HT_2C_ regulation of psychostimulant-mediated behaviors.

## Abbreviations

meth, Methamphetamine; DA, Dopamine; SDZ, SDZ Ser 082; SB206, SB 206553; SB242, SB 242084; 5-HT, Serotonin; CR, Cue reactivity.

## Competing interests

The authors have no competing interests or financial disclosures to report.

## Authors’ contributions

Experiments and data analysis were conducted by SMG. Experimental design and manuscript writing were done by SMG and TCN. All authors read and approved the final manuscript.
